# Endoscopic retrograde submucosal tunnel resection for cervical esophageal submucosal tumors via percutaneous gastrostomy: a conceptual approach

**DOI:** 10.3389/fmed.2025.1651473

**Published:** 2025-10-28

**Authors:** Conghua Song, Xiaomei Li

**Affiliations:** ^1^Gastrointestinal Endoscopy Center, The Affiliated Hospital of Putian University, Putian, Fujian, China; ^2^Key Laboratory of Translational Tumor Medicine in Fujian Province, Putian University, Putian, Fujian, China; ^3^School of Basic Medicine, Putian University, Putian, Fujian, China

**Keywords:** cervical esophagus, submucosal tumor, STER, retrograde endoscopy, percutaneous gastrostomy

## Abstract

Management of submucosal tumors (SMTs) or subepithelial lesions (SELs) at the cervical esophagus remains technically challenging due to limited maneuvering space and short oral mucosal length for conventional submucosal tunnel endoscopic resection (STER). We propose a novel conceptual approach—endoscopic retrograde submucosal tunnel resection (ER-STER)—which enables retrograde access to cervical esophageal SMTs through a percutaneous endoscopic gastrostomy (PG) and anal submucosal tunnel. This technique could potentially enlarge the working space and reduce manipulation near the upper esophageal sphincter (UES), while introducing uncertainties such as PEG-related morbidity, retrograde tunnel perforation, and mediastinal contamination risk. This hypothesis-generating article outlines the rationale, procedural concept, risk analysis, and a translational roadmap for ER-STER. By shifting tunnel entry to the anal side, ER-STER may address the anatomical limitations of conventional STER and reduce patient discomfort associated with proximal mucosal injury. While still theoretical, this method warrants further exploration for feasibility, safety, and clinical utility.

## 1 Introduction

The submucosal tumors (SMTs) or subepithelial lesions (SELs) in the cervical esophagus remains one of the most technically challenging segments for endoscopic resection because of limited working space, acute angulation, and its proximity to the upper esophageal sphincter (UES) (1). At present, clinical evidence concerning endoscopic resection, including ESD, ESE, EFTR, or STER, for cervical esophageal SMTs or SELs is scarce, primarily limited to isolated case reports and small case series (2, 3). These reports frequently describe failed STER attempts due to anatomical constraints, namely a narrow lumen, acute angulation, and a short oral mucosal segment that impede successful tunnel creation and secure closure. Therefore, data on STER outcomes in the cervical esophagus are currently lacking (4–6). By contrast, studies of ESD for mucosal-surface lesions in the cervical esophagus provide indirect insight into these limitations. For instance, the incidence of post-ESD stenosis also tended to be higher in cervical esophagus than the thoracic esophagus (11.6–33.3% vs. 6.6–6.7%), requiring more dilation sessions and longer treatment duration (7–9). When the circumferential mucosal defect exceeded three-quarters, postoperative stricture occurred in 72.7% of patients, with some developing perforation after balloon dilation (10). Even half-circumferential resections markedly increase stricture risk and that steroid injection alone may be insufficient when resection involves ≥ 3/4 of the circumference (11).

Transcervical surgical approaches (12) may overcome some endoscopic limitations but at the cost of external incisions, specialized access, and procedure-related morbidity, while hybrid natural orifice transluminal endoscopic surgery (NOTES) can improve visualization yet still relies on oral-side access and tunneling across the UES. Beyond complication rates, the cervical anatomy (narrow lumen, striated-muscle propulsion, and potential watershed perfusion) magnifies stricture susceptibility, and oral entry or closure near the UES often adds patient discomfort and technical difficulty. These liabilities have driven interest in alternative access strategies that require “out-of-the-box” thinking, novel access routes, and improve working angles and visualization.

Against this backdrop, we posit that a retrograde route to the cervical esophagus via a percutaneous endoscopic gastrostomy (PEG)—termed Endoscopic Retrograde Submucosal Tunnel Resection (ER-STER)—may enlarge the working envelope, optimize endoscopic visualization, and reduce manipulation near the UES by shifting tunnel entry to the anal side. This conceptual pathway is intended to address the specific anatomic limitations of antegrade STER while acknowledging trade-offs introduced by PEG creation (e.g., stoma care and infection risk). This Hypothesis and Theory article delineates the procedural concept, contrasts it against existing options, and outlines a translational roadmap for feasibility testing, with a conceptual comparison summarized in Table 1. While not yet validated in clinical or experimental settings, we present a stepwise framework supported by schematic illustrations and discusses its potential for future application.

**Table 1:** Conceptual comparison of ER-STER versus existing options for cervical esophageal SMTs.

Dimension	Antegrade ESD/STER	Transcervical way	Hybrid NOTES	Proposed ER-STER*
Access route to SMTs	Oral	Cervical	Oral + Cervical	PEG tract+ Anal
Working space	Limited	Good	Mixed	Improved
Visualization/orientation	Challenging near UES	Direct surgical view	Mixed	Improved from anal side
Mucosal entry site	Oral	External	Oral	Aanal side
Patient comfort at UES	Discomfort	Not applicable	Discomfort	Improved
Stricture risk at entry	High	Moderate	High	Low
Perforation risk	Esophageal	Esophageal/surgical site	Mixed	Esophageal+PEG site
Closure complexity	Hard	Mixed	Mixed	Moderate
Infection risk	Low	Moderate	Mixed	Moderate
Logistics/learning curve	Familiar	Requires surgical team	Mixed	Familiar

*Conceptual: requires validation in preclinical and clinical studies.

### 1.1 About the hypothesis and theory of ER-STER

To address these limitations, we propose a conceptual technique—ER-STER. This method employs a retrograde approach, accessing the lesion from the anal side via a PEG and establishing a submucosal tunnel toward the cervical esophagus. Creating the tunnel in an anal→oral direction helps avoid pharyngeal plexus injury and postoperative swallowing discomfort.

The conceptual framework of ER-STER draws from multiple advanced endoscopic procedures, main including:

- NOTES: conceptual precedents for transgastric insertion.
- PEG: providing a practical route for retrograde access.

**Figure 1:**
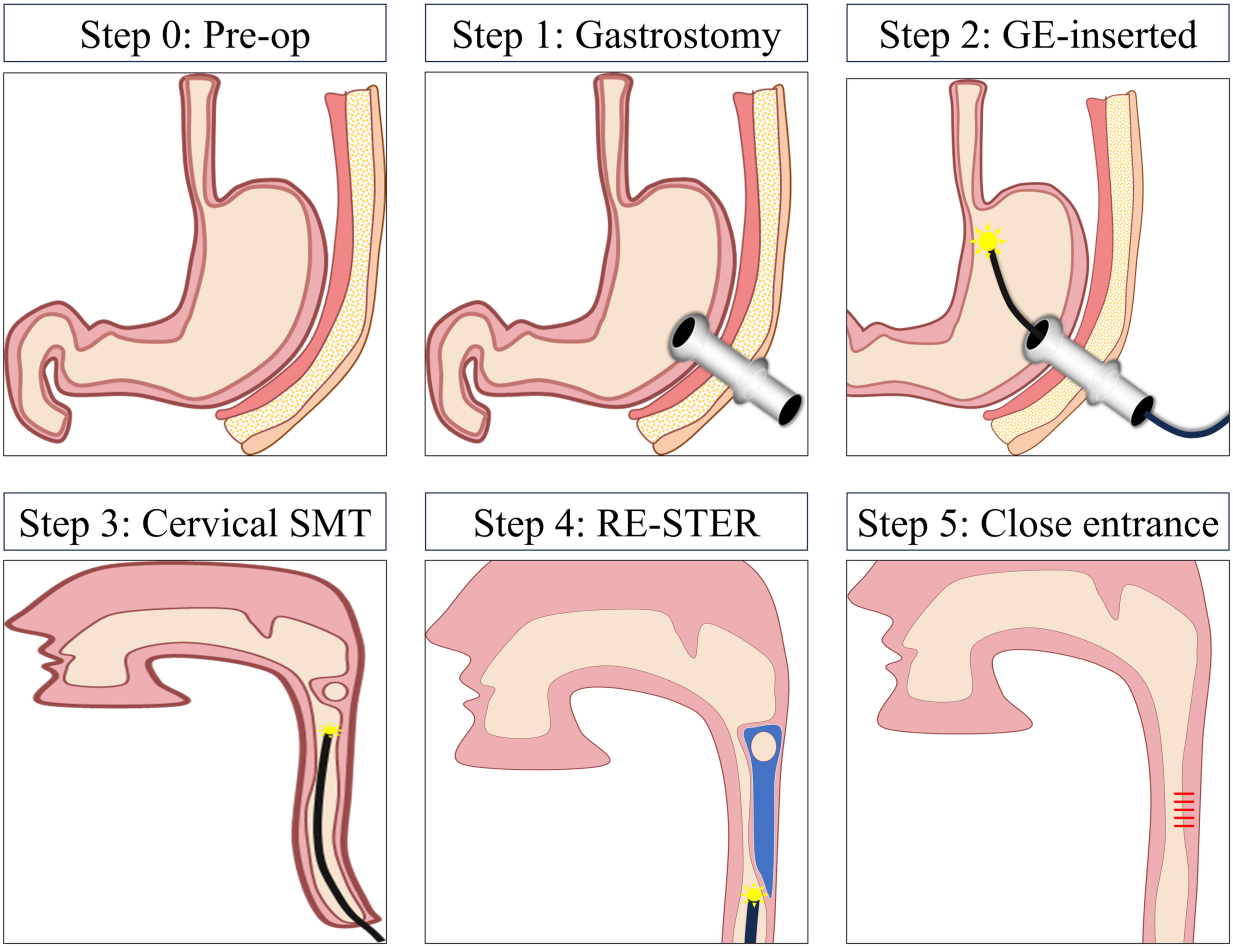
Stepwise schematic of the proposed ER-STER procedure for cervical esophageal SMTs.

SMTs at the cervical esophagus remain technically challenging because of limited working space and short oral mucosa. Conventional STER often proves inadequate in these anatomically constrained areas. ER-STER enables retrograde endoscopic access to the cervical esophagus via a PEG, thereby potentially overcoming these constraints. The procedure consists of six main steps (Figure 1):

- Step 0 (Preoperative evaluation): Imaging and anatomical review to determine feasibility of retrograde access for cervical esophageal SMTs.
- Step 1 (Gastrostomy creation): A PEG is established at the selected puncture site to serve as the transgastric access route.
- Step 2 (Endoscope insertion): The therapeutic gastroscope is inserted through the PEG tract, guided retrogradely to the gastric fundus and then the distal esophagus under direct visualization.
- Step 3 (Cervical lesion localization): The submucosal tumor in the cervical esophagus is identified and marked under direct endoscopic vision.
- Step 4 (ER-STER resection): A submucosal tunnel is created on the anal side of the lesion, approximately 3–5 cm distal to the SMTs, enabling retrograde *en bloc* resection using standard STER knives and lifting solutions. Orientation cues and tunnel markings help maintain directionality.
- Step 5 (Closure of access sites): After lesion removal, the tunnel entry in the esophageal mucosa is closed using endoscopic clips or an over-the-scope device. The endoscope is then withdrawn. The gastric mucosal opening is closed via endoscopic purse-string suturing or other closure systems, and the abdominal wall skin incision is closed using standard surgical techniques.

## 2 Proposed technical detailing

### 2.1 PEG site selection and tract maturation

The PEG insertion site follows standard practice for percutaneous endoscopic gastrostomy. Optimal puncture points are determined using transillumination, finger indentation, and “safe tract” testing. In most patients, the site is chosen in the left upper quadrant, approximately 2–3 fingerbreadths below the left costal margin along the left para-midline, which offers a thinner abdominal wall, a straighter trajectory, and distance from major vessels and organs. The gastric puncture site should avoid the antrum because of frequent peristalsis, which may predispose to tube migration. Preoperative abdominal ultrasound or CT is recommended to rule out interposed colon, ascites, or other anomalies. Patients with coagulopathy, abdominal wall infection, or other contraindications require individualized assessment. In post-gastrectomy patients (e.g., Billroth II reconstruction), puncture is performed closer to the left costal margin but avoiding rib contact to minimize postoperative pain. In cases of abdominal wall scarring, obesity, or organ displacement, imaging guidance should be used to avoid inadvertent injury. Once the tract is established and the PEG bumper secured without leakage on air or water testing, ER-STER may proceed in the same session without delayed maturation.

### 2.2 Endoscope type, cap choice, and distal attachment modifications

A standard therapeutic gastroscope or a newer-generation slim therapeutic scope can be used. A transparent distal attachment—either conventional or tapered—facilitates entry and retrograde navigation. After entering via the PEG, the endoscopist identifies gastric fundus and cardia landmarks, then advances retrogradely to the anal esophagus and up to the cervical segment under direct visualization, typically without the need for additional navigation devices.

### 2.3 Submucosal injection strategy and orientation cues

In ER-STER, the submucosal injection point is shifted from the oral side of the lesion (3–5 cm in conventional STER) to the anal side (3–5 cm beyond the lesion). This distance is gauged by scope markings relative to the PEG entry point. Submucosal lifting is otherwise performed as in conventional STER (e.g., saline-epinephrine-indigo carmine or hyaluronic acid). During tunnel creation, the entry point should be aligned as closely as possible with the longitudinal axis of the SMTs, and the dissection plane kept perpendicular to the circular muscle layer. For endoscopists already proficient in conventional STER, these orientation adjustments are minor and minimize the risk of disorientation.

### 2.4 Maintaining insufflation and preventing leakage

With a properly secured PEG tube, the internal gastric bumper and external skin fixation provide a sealed tract, so significant leakage is unlikely. If minor leakage occurs, it can be managed by routine decompression as in endoscopic full-thickness resection (EFTR). Carbon dioxide insufflation at low pressure is recommended throughout the procedure.

### 2.5 Managing acute retroflexion to reach the cervical esophagus

Preprocedural evaluation with a standard oral gastroscope helps define esophageal course and upper GI anatomy, while review of thoracic CT can identify a narrow thoracic inlet. Retroflexion can be facilitated by patient positioning (left lateral or slight head-up tilt), use of slimmer-diameter scopes, and by careful torque control rather than forceful manipulation. In patients with a severely narrow thoracic inlet, ER-STER should be performed with extreme caution or deferred.

### 2.6 Retrograde navigation and tunnel orientation control

#### 2.6.1 Retrograde passage through the gastroesophageal junction

After PEG creation and insertion of the therapeutic gastroscope, the retrograde passage through the gastroesophageal junction (GEJ) can be facilitated by mild left lateral positioning combined with CO₂ insufflation at ≤6 mmHg to gently distend the lumen without over pressurization. A soft transparent cap (length 4–5 mm) is attached to the scope tip to improve mucosal apposition. Gentle torque and pull-back maneuvers are applied under direct vision, avoiding excessive force on the lower esophageal sphincter. In difficult cases, a guidewire (0.035 inch) inserted from the oral side can serve as an orientation rail for retrograde advancement through the cardia.

#### 2.6.2 Manipulation in the narrow thoracic inlet

In the upper thoracic and cervical segments, the lumen narrows sharply. To enhance maneuverability, the operator may switch to a slim therapeutic gastroscope (outer diameter ≈ 9.2 mm). Patient positioning with slight head elevation (10–15°) can help straighten the esophageal axis. Fine torque steering and gradual deflection rather than pushing force are essential to prevent mucosal trauma. Real-time fluoroscopic monitoring can be employed in preclinical studies to verify scope trajectory and avoid mediastinal deviation.

#### 2.6.3 Orientation and submucosal tunnel guidance

Maintaining correct directionality during retrograde tunneling is critical. Orientation cues include pre-marked submucosal tattooing or endoscopic clips placed orally to the lesion before the retrograde approach. The tunnel should follow the longitudinal axis of the esophagus, verified by observing the muscle fiber orientation. Periodic withdrawal and re-entry allow recalibration of the visual axis.

#### 2.6.4 Submucosal injection and electrosurgical settings

The lifting solution typically consists of 10 mL aliquots of 0.4% sodium hyaluronate + indigo carmine + diluted epinephrine (1:100,000), injected at 5–10 mm intervals to raise the mucosa 5–7 mm. Tunnel creation uses an IT-2 or Dual knife with EndoCut Q mode, effect 3, duration 2, interval 4, and soft coagulation (effect 6, 80 W) for hemostasis. Total injection volume per tunnel segment averages 20–40 mL, depending on length and fibrosis.

In addition, using an electrosurgical knife equipped with a water-jet or injection-assisted function (e.g., FlushKnife BT or HybridKnife) can accelerate tunnel creation by simultaneously maintaining mucosal lifting and cutting efficiency, thereby reducing procedure time and improving field visibility.

## 3 Risk analysis

### 3.1 Anticipated stricture risk based on analogous resections

Conventional STER for submucosal tumors of the esophagus differs fundamentally from ESD because the mucosal layer overlying the lesion is typically preserved. Only a small longitudinal mucosal incision (generally <1.5 cm) is created to establish the tunnel entry. Such small entry sites—particularly when located on the anal side of the lesion, closer to the upper thoracic esophagus—carry a negligible risk of clinically significant stricture. Even if stricture develops, endoscopic balloon dilation can be readily performed. Moreover, because the mucosa overlying the tumor remains intact after *en bloc* submucosal dissection, there is no circumferential denudation comparable to ESD.

### 3.2 Mitigation strategies for stricture

Given the intact mucosal covering in both conventional STER and the proposed ER-STER, large circumferential mucosal defects are unlikely. Therefore, prophylactic steroid injections or tissue-shielding measures (e.g., polyglycolic acid sheets) are generally unnecessary. Standard closure of the small tunnel entry with clips, as in STER, should suffice. Nonetheless, minimizing the mucosal entry arc—ideally <50% of the circumference—remains prudent to further reduce stricture risk and facilitate secure closure.

### 3.3 PEG-related morbidity, retrograde tunnel perforation, and mediastinal contamination

PEG placement is a mature, widely used procedure with well-characterized and generally low complication rates. As in standard PEG practice, careful site selection with transillumination, safe tract testing, and imaging guidance minimizes stoma-related complications. For ER-STER, experienced operators can first perform a standard oral gastroscopy to familiarize themselves with upper GI anatomy, then switch to retrograde entry via the PEG tract. Recognizing key anatomic landmarks such as the fundus and cardia allows straightforward navigation to the esophageal inlet. This, combined with low-pressure CO₂ insufflation and careful dissection, substantially limits the risk of retrograde tunnel perforation. In the unlikely event of a small perforation, standard endoscopic closure (clips or over-the-scope devices) can be applied. Because the mucosal entry and the submucosal tunnel are spatially distinct from the PEG site, the chance of mediastinal contamination is low. Nonetheless, peri-procedural antibiotics and vigilant monitoring are advisable in translational studies to address this potential risk.

## 4 Translational validation pathway

As ER-STER is currently a conceptual approach, a staged validation pathway is essential to ensure safety and feasibility before any clinical adoption. We envisage the following steps:

Phase 1 – Cadaveric or *ex vivo* porcine model testing: Initial ergonomics, scope maneuverability, and tunnel stability will be refined in cadaveric or *ex vivo* porcine esophagus–stomach blocks, allowing iterative adjustment of PEG placement, tunnel trajectory, and closure techniques under controlled conditions.

Phase 2 – *In vivo* animal studies: Once procedural steps are optimized, *in vivo* animal models (e.g., porcine or canine) would be used to assess healing dynamics, stricture rates at tunnel entry, and infection or leakage risks at both esophageal and PEG sites. This phase will also test different closure and tissue-shielding methods to minimize complications.

Phase 3 – Defined feasibility and safety endpoints: For feasibility, endpoints such as *en bloc* resection rate, procedural time, and completion without conversion will be recorded. For safety, rates of perforation, leakage, bleeding, and stricture will be systematically measured, along with histologic assessment of healing at the tunnel entry. These metrics will inform whether ER-STER meets predefined thresholds to justify pilot human trials.

This stepwise translational roadmap mirrors established pathways for innovative endoscopic procedures and provides a concrete plan to test and refine ER-STER before clinical use. While the present article remains conceptual, preparatory steps for validation are already under design. Phase 1 cadaveric evaluations will assess scope ergonomics and tunnel orientation feasibility, followed by *in vivo* animal studies to analyze healing dynamics and leakage risk. Subsequently, a pilot clinical feasibility trial is being planned at our institution to assess safety, procedural success rate, and short-term postoperative recovery. Ethical approval and protocol development are underway. These progressive stages will generate quantitative data to verify the safety and practical utility of ER-STER before any broader application.

To ensure standardized evaluation across future validation phases, we propose a defined set of outcome metrics. Primary technical endpoints will include: (1) total procedure time, (2) en bloc resection rate, and (3) conversion rate to alternative approaches. Safety endpoints will cover: (1) intra- and postoperative perforation, (2) bleeding requiring intervention, (3) infection or mediastinal leakage, and (4) PEG-site complications. Long-term follow-up indicators will comprise: (1) incidence and severity of esophageal stricture, (2) recurrence or residual submucosal lesion at the resection site, and (3) durability of closure and mucosal healing at 3-, 6-, and 12-month intervals. These predefined metrics will facilitate reproducibility, enable inter-study comparison, and provide quantitative evidence for the clinical translation of ER-STER.

## 5 Discussion

Conventional antegrade approaches for cervical esophageal lesions—particularly ESD and, to a lesser extent, STER—carry nontrivial adverse event profiles amplified by the anatomic constraints of the cervical esophagus (13–15). In ESD, pooled estimates place bleeding at ~2% and perforation at ~3–5%, with large national series reporting perforation near 3%; importantly, post-ESD stricture risk in the cervical segment is disproportionately high and rises steeply when circumferential mucosal defects exceed ~75%, yet clinically relevant strictures can still occur below that threshold (11). By contrast, STER generally preserves the overlying mucosa and thus has low stricture propensity; its adverse events are dominated by gas-related complications (pneumothorax, pneumoperitoneum, mediastinal/pleural effusions) and fever, with most cases managed conservatively. Major bleeding is uncommon (~1–2% in large series), while clinically significant perforation is infrequent but possible (16).

Recent guidelines and conference discussions echo these needs. The ASGE ESD guideline emphasizes careful patient and technique selection due to bleeding, perforation, and stenosis risks in esophageal ESD (17). Meanwhile, Digestive Disease Week (DDW) 2023–2024 sessions specifically reviewed stricture formation and prevention after esophageal ESD, including limits of steroid monotherapy for extensive defects and exploration of tissue-shielding (e.g., polyglycolic acid sheets), underscoring the field’s pursuit of strategies that minimize oral mucosal injury (18).

From an innovation standpoint, the ER-STER concept does not introduce a novel endoscopic device or dissection instrument. This conceptual procedure integrates principles of retrograde access, NOTES, and STER to overcome anatomical limitations in the cervical esophageal SMTs not amenable to conventional oral-side STER. Compared with conventional oral-side STER, ER-STER bypasses the limitations of the proximal esophagus, offering improved visualization, access, and working space. Moreover, it potentially reduces patient discomfort related to mucosal entry near the UES. The essential innovation is the reconfiguration of the access route, transforming a traditionally antegrade tunnel into a retrograde pathway via PEG, thereby expanding the working envelope while maintaining submucosal tunneling principles.

By avoiding oral-side tunneling, ER-STER circumvents limited working space and difficult orientation in the cervical esophagus. Moreover, by shifting mucosal incision to the anal side, ER-STER may mitigate patient discomfort and reduce postprocedural complications. Conceptually, ER-STER could help overcome some anatomic and spatial limitations of oral-side tunneling in the cervical esophagus. However, these potential procedural advantages remain theoretical and have not yet been validated in preclinical or clinical settings. Any reduction in patient discomfort or complication rates must be verified empirically through controlled feasibility and safety studies. Moreover, potential risks, including retrograde navigation difficulty, anastomotic leakage, and infection at the PEG site, require careful assessment.

ER-STER should be regarded as a conceptual framework intended to stimulate further exploration and discussion within the endoscopic community. ER-STER remains at a purely conceptual and hypothesis-generating stage. No animal or human feasibility data currently exist to support its safety, efficacy, or procedural reproducibility. The proposed benefits, such as improved access, enhanced visualization, and reduced mucosal tension near the UES, should therefore be interpreted as speculative working hypotheses.

Nonetheless, we believe this innovative concept may offer a promising alternative for managing otherwise inoperable cervical esophageal SMTs and invite further discourse on its feasibility to inspire future technical innovations in transluminal endoscopy. Future studies are needed to evaluate the technical feasibility, safety, and potential indications of ER-STER in clinical practice.

## Data Availability

The original contributions presented in the study are included in the article/supplementary material, further inquiries can be directed to the corresponding authors.
